# Application of Machine Learning Methods to Ambulatory Circadian Monitoring (ACM) for Discriminating Sleep and Circadian Disorders

**DOI:** 10.3389/fnins.2019.01318

**Published:** 2019-12-10

**Authors:** Beatriz Rodriguez-Morilla, Eduard Estivill, Carla Estivill-Domènech, Javier Albares, Francisco Segarra, Angel Correa, Manuel Campos, Maria Angeles Rol, Juan Antonio Madrid

**Affiliations:** ^1^Laboratory of Chronobiology, IMIB-Arrixaca, Department of Physiology, Centro de Investigación Biomédica en Red de Fragilidad y Envejecimiento Saludable, Instituto de Salud Carlos III, University of Murcia, Murcia, Spain; ^2^Clínica del Sueño Estivill, Barcelona, Spain; ^3^Fundación Estivill Sueño, Barcelona, Spain; ^4^Medicina del Sueño Doctor Albares, Centro Médico Teknon, Barcelona, Spain; ^5^Department of Experimental Psychology, Faculty of Psychology, University of Granada, Granada, Spain; ^6^Department of Computing and Systems, Faculty of Computer Science, University of Murcia, Murcia, Spain

**Keywords:** circadian rhythms, wrist temperature, actigraphy, light exposure, insomnia, delayed sleep phase, decision tree, digital health

## Abstract

The present study proposes a classification model for the differential diagnosis of primary insomnia (PI) and delayed sleep phase disorder (DSPD), applying machine learning methods to circadian parameters obtained from ambulatory circadian monitoring (ACM). Nineteen healthy controls and 242 patients (PI = 184; DSPD = 58) were selected for a retrospective and non-interventional study from an anonymized Circadian Health Database (https://kronowizard.um.es/). ACM records wrist temperature (T), motor activity (A), body position (P), and environmental light exposure (L) rhythms during a whole week. Sleep was inferred from the integrated variable TAP (from temperature, activity, and position). Non-parametric analyses of TAP and estimated sleep yielded indexes of interdaily stability (IS), intradaily variability (IV), relative amplitude (RA), and a global circadian function index (CFI). Mid-sleep and mid-wake times were estimated from the central time of TAP-L5 (five consecutive hours of lowest values) and TAP-M10 (10 consecutive hours of maximum values), respectively. The most discriminative parameters, determined by ANOVA, Chi-squared, and information gain criteria analysis, were employed to build a decision tree, using machine learning. This model differentiated between healthy controls, DSPD and three insomnia subgroups (compatible with onset, maintenance and mild insomnia), with accuracy, sensitivity, and AUC >85%. In conclusion, circadian parameters can be reliably and objectively used to discriminate and characterize different sleep and circadian disorders, such as DSPD and OI, which are commonly confounded, and between different subtypes of PI. Our findings highlight the importance of considering circadian rhythm assessment in sleep medicine.

## Introduction

The study of circadian rhythms, especially when applicable to clinical practice, has generated increasing interest over the last few years (Sadeh and Acebo, 2002; Ancoli-Israel et al., 2003). This is partially due to lifestyle changes in developed societies that directly affect the quality of circadian rhythms, with the subsequent impact on sleep (Shochat, 2012). As reviewed by Goel et al. (2014), this so-called *24/7 society* is characterized by an increasing proportion of work and leisure activities at night, exposure to light at aberrant times of the day, growing use of technological devices, and *social jetlag*, i.e., a circadian misalignment between free and work days (Roenneberg et al., 2003). All these factors lead to desynchronization between internal rhythms and the environmental day–night cycle. Moreover, the use of light-emitting electronic devices at night has been associated with delayed, reduced, and fragmented sleep (Owens et al., 1999; Van Den Bulck, 2004; Shochat et al., 2010).

In turn, sleep disorders usually lead to circadian disruption. On the one hand, sleep timing and duration modulate exposure to environmental cues that synchronize the circadian system (e.g., environmental light, food schedules, social activities, etc.). On the other hand, common symptoms of sleep disorders such as sleep fragmentation or short sleep duration, apart from contributing to excessive daytime sleepiness, affect the amplitude of other rhythms (Martinez-Nicolas et al., 2011) and alter the balance between homeostatic and circadian processes involved in sleep–wake regulation (Borbély et al., 2016).

This bidirectional relationship between sleep and circadian alterations highlights why evaluating circadian rhythms is also important for sleep medicine. In the current study, we aimed to test the diagnostic usefulness of ACM, based on wearable technology which combines the simultaneous recording of several circadian output and input signals, namely wrist temperature (T) (Sarabia et al., 2008), motor activity (A), body position (P) (Sadeh and Acebo, 2002; Ancoli-Israel et al., 2003), and exposure to environmental light (L) rhythms. Actigraphy is accepted by the American Academy of Sleep Medicine (AASM) as clinically appropriate for studying sleep and circadian disorders, as it has been used for >20 years (Sadeh and Acebo, 2002; Ancoli-Israel et al., 2003). In addition, wrist temperature has been shown as a reliable circadian marker (Sarabia et al., 2008; Martinez-Nicolas et al., 2013), showing a close relationship with dim light melatonin onset (Bonmatí-Carrión et al., 2014) and being therefore proposed as a non-intrusive circadian marker of choice in a consensus document (Mullington et al., 2016). Its usefulness for diagnostic purposes has been also reported for multiple conditions (Zornoza-Moreno et al., 2013; Martinez-Nicolas et al., 2014, 2018). The variables T, A, and P can be combined into the integrated variable TAP, which indicates general activation and has been previously validated for estimating sleep, showing greater reliability than any of the individual variables by themselves according to both sleep diaries (Ortiz-Tudela et al., 2010) and polysomnographic assessment (Ortiz-Tudela et al., 2014). Recently, this method has been successfully employed for chronotype identification (Martinez-Nicolas et al., 2019). In the present study, the usefulness of ACM as a diagnostic tool has mainly focused on PI and DSPD, due to their high prevalence and the overlapping of their symptoms (Gradisar and Crowley, 2013; Sivertsen et al., 2013).

Insomnia may be both a symptom and a disorder. Briefly, it implies difficulty for initiating or maintaining sleep, or non-restorative sleep, when a favorable opportunity and circumstances arise. It is considered a disorder when these symptoms are present at least three times a week, for at least 1 month (Roth, 2007). As such, it is the most common sleep disorder, but its prevalence varies depending on the definition used and the population studied. The strictest diagnostic criteria suggest a prevalence rate of around 5–7%, although insomnia symptoms rise to nearly 30% (Roth, 2007).

Delayed sleep phase disorder refers to normal sleep (in terms of quality and structure) that shows significantly delayed onset and wake-up times, with respect to those externally imposed, or desired by the patient according to standard schedules (American Academy of Sleep Medicine, 2005; World Health Organization [WHO], 2008). This also implies difficulties for initiating sleep, as in the case of insomnia, as well as problems for waking up early in the morning. The accomplishment of standard wake times results in partial sleep deprivation, which in turn deteriorates daytime performance (Gradisar and Crowley, 2013). Studies on the general population yielded prevalence rates of between 0.13 and 0.17% (Schrader et al., 1993; Yazaki et al., 1999), but when focusing on adolescence, prevalence increases to 16% (Lovato et al., 2013). Nevertheless, all estimations until now are uncertain, since this disorder is likely underestimated (Gradisar et al., 2011) and probably confounded with OI (Weitzman et al., 1981). Thus, tools are needed that allow their differentiation, in order to select the most appropriate interventions.

Prediction and classification methods based on machine learning are rapidly expanding as diagnostic tools for a wide spectrum of pathologies (Kubota et al., 2016; Dagliati et al., 2017; Duda et al., 2017; Kim et al., 2017; Mossotto et al., 2017; Serrano et al., 2017). The aim of this field of computational sciences is to develop algorithms capable of detecting predictable patterns in complex data samples. In general terms, supervised machine learning permits classification when the output is a label or nominal value, and prediction when the output is a numeric value. In this study, we used a classification model known as decision tree: a top-down classification algorithm that splits data into hierarchical nested classes, by selecting the variable in each step that better subdivides the sample (Rokach and Maimon, 2005). Comparing the classification obtained with an expert criterion (in this case, the initial categories of pathologies) allows estimating both the sensitivity and specificity of the generated model (Kubota et al., 2016).

Thus, the aim of this study was to assess the usefulness of machine learning methods to differentially diagnosis PI from DSPD using the circadian rhythms of wrist temperature, motor activity, body position, and exposure to environmental light, recorded by a ACM device.

## Materials and Methods

### Participants

A total of 242 patients suffering from either PI (*n* = 184) or DSPD (58), and 19 healthy control subjects were included in this study. Patients and healthy controls were retrospectively selected from the Circadian Health Database of the Chronobiology Laboratory at the University of Murcia^1^. Chronobiological and sleep parameters from subjects attendants to Dr. Estivill Sleep Clinic (Barcelona, Spain) were anonymously analyzed and coded by the Kronowizard platform which was approved by the Ethics Committee of the University of Murcia. All subjects gave written informed consent before their diagnosis tests according to the model provided in the Kronowizard platform. The selection criteria for patients were: (1) having been diagnosed of either PI or DSPD by sleep experts from the above mentioned sleep clinic, (2) including ACM as part of routine clinical diagnosis and treatment of sleep and circadian disorders, (3) not being diagnosed of any other primary circadian or sleep disorders (discarded by PSG). The available information from the database did not differentiate between onset, maintenance, or terminal insomnia. The selection criterion for healthy controls was not being diagnosed of any sleep or circadian disorder. Exclusion criteria for both patients and healthy controls were any organic, metabolic, endocrine, or psychiatric disorder, so as the abuse of alcohol or illicit drugs.

### Clinical Interview

The diagnostic classification was based on the clinical history. To this end, patients were interviewed about the nature of their complaints, including questions aimed at addressing the following aspects:

– Whether the problem referred to sleep onset, maintenance, non-restorative sleep, or a combination of these.
– Duration, frequency, and severity of the symptoms.
– Daytime functioning and associated symptoms.
– Other possible symptoms (snoring, apnea, nocturia, parasomnias, symptoms associated with other disorders, such as restless legs syndrome, periodic limb movement disorder, etc.).
– Sleep–wake habits: work, school, food and social schedules, physical activity, use of technological devices at night, naps during daytime, caffeine intake, routines before bedtime, etc.
– Sleep conditions and routines: conditions of the bedroom (light, noise, etc.), whether the patient sleeps alone or with someone else (and, in this case, possible disturbances from the bedmate), the presence and use of technological devices once in bed (TV, computer/laptop, light-emitting e-books, radio, etc.).
– Time preferences for sleep onset, wakeup, activities schedule, etc.
– Intake of psychoactive substances (alcohol, stimulating or relaxing substances, legal drugs, illicit drugs, etc.).

### Ambulatory Circadian Monitoring Device

**FIGURE 1:**
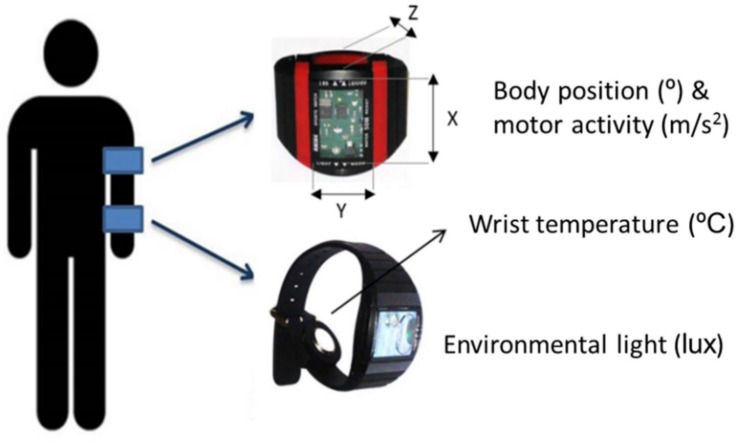
Schematic arrangement of the ambulatory circadian monitoring device Kronowise^®^, composed of a luxometer and a temperature data logger placed on the wrist, and an accelerometer placed on the arm (Chronobiology Laboratory, University of Murcia).

The multichannel device Kronowise^®^ (Chronobiology Laboratory, University of Murcia, Murcia, Spain) employed to evaluate the circadian rhythms of the selected sample integrates several sensors: (1) The Thermochron^®^ iButtonDS1921H data logger (Dallas, Maxim), placed on the skin of the non-dominant wrist, assesses distal temperature every 10 min; (2) an actimeter (Hobo^®^ Pendant G Acceleration Data Logger), placed on the non-dominant arm, records body position as the tilt (°) of the vertical axis parallel to the arm (axis *X*, Figure 1) and motor activity as its acceleration (m/seg^2^), every 30 s; and (3) a luxometer (Hobo^®^ Pendant Light-Temperature Data Logger) registers environmental light (in luxes) every 30 s. All subjects wore this equipment 24 h a day for a week, including work and non-work days, under normal-living conditions as already described (Refinetti et al., 2013; Martinez-Nicolas et al., 2019). During the registration period, participants were instructed to follow their usual lifestyle: since sleep assessment was performed within a clinical context, the ACM study was intended to be conducted in conditions as faithful as possible to their normal living at the moment of their complaints.

### Data Analysis

#### Non-parametric Analysis

The integrated variable TAP was calculated for each subject, from his/her rhythms of skin temperature, motor activity, and body position, as described in Ortiz-Tudela et al. (2010). To this end, the values of skin temperature were normalized between 0 and 1 using the 5th and 95th percentile and inverted; the values of motor activity were normalized with respect to the actimeter placement. Normalized activity values ranged from 0 (total immobility) to 1 (percentile 95th, used as maximal acceleration reference). Arm position was also normalized between 0 (horizontal) to 1 (vertical position). The integrated variable TAP expressed the level of general activation from 0 (minimal activation, high temperature, low activity, and horizontal arm position) to 1 (maximal activation, low temperature, high activity, and vertical arm position). This was used to estimate sleep probability, from a threshold dynamically established for each subject, depending on his/her own rhythms (Ortiz-Tudela et al., 2010).

All these variables, including TAP and estimated sleep, were subsequently subjected to non-parametric analyses (Madrid-Navarro et al., 2019) using the software Circadianware^®^ implemented in the website Kronowizard^®2^ (Chronobiology Laboratory, University of Murcia, Murcia, Spain), yielding the following indexes:

• M10v and M5v, and L10v and L5v refer to the mean values of 10 or 5 consecutive hours of maximal (M) and lowest (L) values of the variable. M5v of temperature and L5v of activity, position, and TAP have been used as indexes of sleep depth, while L10v of temperature and M10v of activity, position, and TAP indicate general activation levels during wakefulness. The midpoint timing of these periods (M10h and M5h, and L10h and L5h) was also obtained and used as indexes of circadian phase.
• IS quantifies the repetitiveness of the rhythm across consecutive days. It is obtained according to the following formula:

$$\text{IS}=\frac{n\,\sum_{h=1}^{n}{\left({\bar{x}}_{h}-\bar{x}\right)}^{2}}{p\,\sum_{i=1}^{n}{\left(x_{i}-\bar{x}\right)}^{2}}$$

*n*: total number of data,
  *p*: number of data per day,
  $\bar{x}$: mean value of all the data,
${\bar{x}}_{h}$: mean value of the data at a specific time of day,
IS ranges from 0 (maximum noise) to 1 (perfect IS, i.e., when the daily wave repeats exactly across days).

Intradaily variability indicates the rhythm fragmentation, which depends on the frequency and extension of the transitions between low and high values within the cycle, according to the following formula:

$$\text{IV}=\frac{n\,\sum_{i=2}^{n}{\left(x_{i}-x_{i-1}\right)}^{2}}{\left(n-1\right)\,\sum_{i=1}^{n}{\left(x_{i}-\bar{x}\right)}^{2}}$$

*n*: total number of data,
$\bar{x}$: mean value of all the data.

Intradaily variability values are close to 0 in the case of a perfect sinusoid wave, and approach 2 in the case of Gaussian noise.

• *Relative amplitude* is a marker of the rhythm amplitude or contrast between wakefulness and sleep values, i.e., the difference between the maximum and minimum values, according to the following formula for motor activity, body position, TAP, and environmental light:

$$\text{RA}=\frac{\mathrm{M10v}-\mathrm{L5v}}{\mathrm{M10v}+\mathrm{L5v}}$$

As wrist temperature and estimated sleep show opposite profiles, RA was calculated as follows:

$$\text{RA}=\frac{\mathrm{M5v}-\mathrm{L10v}}{\mathrm{L10v}+\mathrm{M5v}}$$

The RA for WT was multiplied by 10 in order to amplify its values to range from 0 to 1. Values near 0 in this index indicate null contrast between wakefulness and sleep, while values near 1 express maximal contrast.

• The CFI was developed to characterize rhythm robustness with a single score (Ortiz-Tudela et al., 2010). It integrates normalized values between 0 and 1 for IS, IV, and RA, with IV values being inverted. Accordingly, the CFI ranges from 0 (null circadian rhythmicity) to 1 (maximally robust circadian rhythm).

#### Machine Learning Analysis

All subjects included in our study were classified by machine learning analysis, using a decision tree, performed on the indexes described above. This analysis was carried out using the software Orange Canvas© (Demšar et al., 2013).

##### Attribute selection

The selection of attributes for the classification model was based on the TAP variable and sleep probability estimated from it, due to its greater validity over the individual variables (Ortiz-Tudela et al., 2010, 2014). Attribute selection was guided by the expert criterion of including those indexes providing complementary information to one another. Therefore, we aimed to select indexes describing circadian phase and rhythm and sleep quality.

With this in mind, the discriminative potential of the candidate attributes was obtained according to the criterion of information gain (based on entropy reduction) and the statistical criteria of ANOVA (maximization of differences between classes) and Chi-squared (maximization of internal correlation within each class) measures.

##### Discretization

The selected attributes were reconverted from continuous to discrete values, since classification models built on discrete attributes are more exact than those built on continuous ones (Demšar et al., 2013). Discretization was based on data splits that met the criteria proposed by Maslove et al. (2013) and Liu and Hussain (2002): (1) reflecting the original distribution of the continuous attribute; (2) maintaining the attribute patterns without adding additional spurious patterns; and (3) making sense and being interpretable according to expert criteria.

The discretization method used in our study was minimum description length (MDL) (Fayyad and Irani, 1993). This is a top-down technique that recursively splits the attribute maximizing information gain to the point where a new split would not add any new information to the predictions.

##### Model validation

The model was evaluated through 10-fold cross-validation, obtaining the following indexes:

• Sensitivity, true positive rate, or recall: probability of correctly classifying a case. It is obtained according to this formula:

$$\text{Sensitivity}=\frac{\text{TP}}{\text{TP}+\text{FN}}$$

TP: number of true positives,

FN: number of false negatives.

• Accuracy, predictive value: probability of a specific case actually belonging to the class assigned by the model, according to the following formula:

$$\text{Accuracy}=\frac{\text{TP}}{\text{TP}+\text{FP}}$$

TP: number of true positives,

FP: number of false positives.

• F1 score indicates sensitivity and accuracy together, as the harmonic mean of the two, i.e.,

$$\text{F}\,1=2\,\frac{\text{Sensitivity}\,x\,\text{Accuracy}}{\text{Sensitivity}+\text{Accuracy}}$$

• Specificity or true negative rate is the probability of correctly excluding a case from a class where it does not belong, obtained as follows:

$$\text{Specificity}=\frac{\text{TN}}{\text{TN}+\text{FP}}$$

TN: number of true negatives,
FP: number of false positives.
• False positive rate: probability of erroneously assigning a case to a class where it does not belong, calculated as:

False⁢positives⁢rate=1-Specificity

• *Receiver operating characteristic* (ROC) curve and AUC: the ROC curve is obtained from the graphic representation of sensitivity and false positives rate together. The AUC is a discriminating measure that indicates the capacity of the model to discriminate values of different classes.

## Results

### Non-parametric Analyses

The circadian markers obtained from ACM and the circadian indexes obtained from non-parametric analyses for every original class (i.e., diagnostic categories based on the clinical history previous to machine learning classification) are summarized in Table 1.

**TABLE 1:** Mean value (standard deviations in brackets) for every index obtained from non-parametric analysis for wrist temperature, motor activity, TAP, estimated sleep, and environmental light.

**Circadian marker/index**	**Temperature**	**Act**	**TAP**	**Sleep**	**Light**
**L5v/M5v**	**(°C)**	**Norm. (0–1)**	**A.U.**	**Probability**	**Log(lux)**
Control	34.92 (0.54)	0.04 (0.02)	0.13 (0.02)	0.95 (0.03)	0.01 (0.04)
Insomnia	34.54 (0.86)	0.06 (0.06)	0.18 (0.06)	0.9 (0.07)	0.03 (0.08)
DSPD	34.35 (0.87)	0.08 (0.07)	0.19 (0.06)	0.87 (0.09)	0.11 (0.19)

**L5h/M5h**	**hh:mm**	**hh:mm**	**hh:mm**	**hh:mm**	**hh:mm**

Control	4:04(0:51)	3:57(0:36)	3:54(0:42)	4:06(0:22)	3:52(0:38)
Insomnia	4:44(4:24)	3:54(1:14)	4:04(1:56)	3:57(1:10)	3:54(2:26)
DSPD	7:21(3:25)	6:43(2:03)	6:48(1:43)	6:37(1:43)	6:08(2:58)

**M10v/L10v**	**(°C)**	**Norm. (0–1)**	**A.U.**	**Probability**	**Log(lux)**

Control	32.04 (0.84)	0.70 (0.18)	0.63 (0.05)	0.01 (0.02)	1.81 (0.51)
Insomnia	32.46 (1.09)	0.63 (0.24)	0.59 (0.07)	0.05 (0.05)	1.64 (0.56)
DSPD	32.44 (1.26)	0.67 (0.22)	0.58 (0.07)	0.06 (0.07)	1.35 (0.46)

**M10h/L10h**	**hh:mm**	**hh:mm**	**hh:mm**	**hh:mm**	**hh:mm**

Control	15:40(1:39)	16:19(1:32)	16:16(1:04)	15:51(1:54)	14:56(1:40)
Insomnia	14:25(2:37)	15:26(1:56)	15:25(1:47)	15:19(1:52)	14:20(1:41)
DSPD	18:01(3:00)	17:45(3:43)	18:07(3:08)	17:05(5:25)	16:03(3:02)
**RA (relative amplitude) (A.U. 0–1)**					
Control	0.43 (0.16)	0.69 (0.19)	0.66 (0.07)	0.98 (0.03)	0.99 (0.04)
Insomnia	0.31 (0.15)	0.66 (0.16)	0.53 (0.11)	0.89 (0.11)	0.96 (0.11)
DSPD	0.29 (0.17)	0.6 (0.16)	0.5 (0.13)	0.87 (0.13)	0.88 (0.18)
**IS (interdaily stability) (A.U. 0–1)**					
Control	0.52 (0.13)	0.28 (0.04)	0.59 (0.08)	0.75 (0.06)	0.52 (0.1)
Insomnia	0.37 (0.19)	0.28 (0.08)	0.49 (0.12)	0.64 (0.12)	0.48 (0.15)
DSPD	0.32(0.19)	0.26 (0.12)	0.44 (0.16)	0.57 (0.15)	0.4 (0.16)
**IV (intradaily variability) (A.U. 0–2)**					
Control	0.12 (0.05)	0.97 (0.08)	0.3 (0.07)	0.19 (0.05)	0.19 (0.06)
Insomnia	0.16 (0.08)	0.98 (0.09)	0.48 (0.17)	0.32 (0.1)	0.21 (0.15)
DSPD	0.15 (0.08)	1.01 (0.1)	0.48 (0.16)	0.3 (0.1)	0.2 (0.09)
**CFI (circadian functioning index) (A.U. 0–1)**					
Control	0.5 (0.05)	0.54 (0.04)	0.7 (0.05)	0.88 (0.03)	0.8 (0.04)
Insomnia	0.44 (0.07)	0.51 (0.06)	0.59 (0.09)	0.79 (0.08)	0.78 (0.08)
DSPD	0.42 (0.07)	0.49 (0.08)	0.57 (0.11)	0.76 (0.1)	0.73 (0.1)

*M5v/L5v corresponds to the mean values of the five consecutive hours of maximal (M) and lowest (L) values; M10v/L10v, mean values of the 10 consecutive hours of maximal (M) and lowest (L) values; M5h/L5h is the midpoint timing of the five consecutive hours of maximal (M) and lowest (L) values; M10h/L10h, midpoint timing of the 10 consecutive hours of maximal (M) and lowest (L) values. DSPD, delayed sleep phase disorder. A.U. = arbitrary units.*

### Attribute Selection

The attributes selected for building the decision tree classification model, according to the criteria of information gain, ANOVA, and Chi-squared were: TAP-L5h as sleep phase marker, TAP-M10h as wakefulness phase marker, CFI of estimated sleep as an indicator of global quality of the rhythm, and TAP-RA, which apart from being an index for rhythm quality, provides information relative to sleep depth and general activation during wakefulness together. Table 2 shows the magnitudes of the criteria used for each of the selected attributes.

**TABLE 2:** Information gain, ANOVA *F*, and χ^2^ for every attribute selected for building the decision tree (see Figure 1 for legend).

**Attribute**	**Information gain**	**ANOVA (*F*)**	**χ^2^**
TAP-L5h	0.36	50.30	79.06
TAP-M10h	0.25	35.70	53.64
Sleep-CFI	0.13	12.83	25.59
TAP-RA	0.10	13.86	24.08

### Decision Tree

**FIGURE 2:**
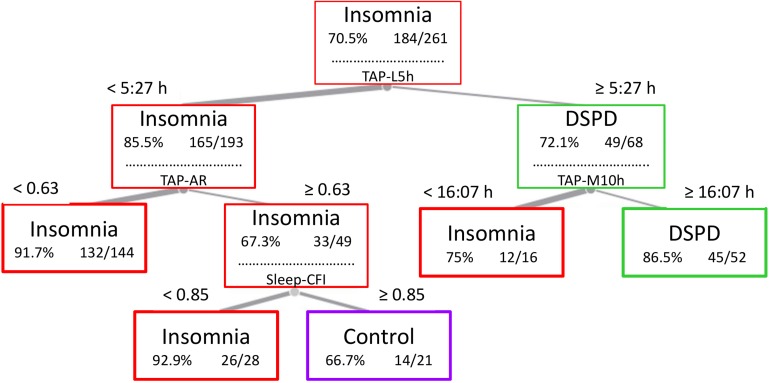
Decision tree and cut values used by the model to generate five final classes: DSPD (with both delayed mid-sleep and central time of maximal activation), OI (delayed mid-sleep time, but not central time of maximal activation), MI (normal sleep phase and low relative amplitude and CFI), mild insomnia (which only differed from the controls in terms of their sleep rhythm robustness), and healthy controls. TAP-L5h, midpoint timing of the five consecutive hours of lowest TAP values; TAP-M10h, midpoint timing of the 10 consecutive hours of maximal TAP values; TAP-AR, relative amplitude of TAP rhythm; Sleep CFI, circadian function index of sleep rhythm.

Subjects were classified as shown in Figure 2, based on the attributes described above. The sleep phase marker TAP-L5h (a marker of mid-sleep time) allows for discriminating between pathologies characterized by sleep onset problems (TAP-L5h later than 5:27 h) and the remaining classes. Subjects with earlier TAP-L5h were then divided according to their TAP-RA, i.e., the contrast between rest and activity period levels. Those with lower TAP-RA (<0.629), classified by the model as insomnia, could be considered as a MI subtype according to its characteristics (explained below). Next, subjects with higher levels of TAP-RA were divided according to their sleep-CFI. This resulted in a group with higher scores (≥0.852), indicating more robust rhythms; these were classified as healthy controls, while a group with lower rhythm robustness, classified by the model as insomnia, could be considered a mild type. On the other hand, the group characterized by a delay in mid-sleep time was subsequently divided according to their TAP-M10h, i.e., the central time of maximal activation. This allowed for differentiating between DSPD (TAP-M10h later than 16:07 h) and a third group of insomnia (TAP-M10 earlier than 16:07 h) compatible with an insomnia onset subtype. In summary, this model generated five final classes: healthy controls, DSPD (with both delayed mid-sleep and central time of maximal activation), and three subtypes of insomnia, henceforth referred to as OI (delayed mid-sleep time, but not central time of maximal activation), MI (normal sleep phase and low RA and CFI), and mild insomnia (which only differed from the controls in terms of their sleep rhythm robustness).

**FIGURE 3:**
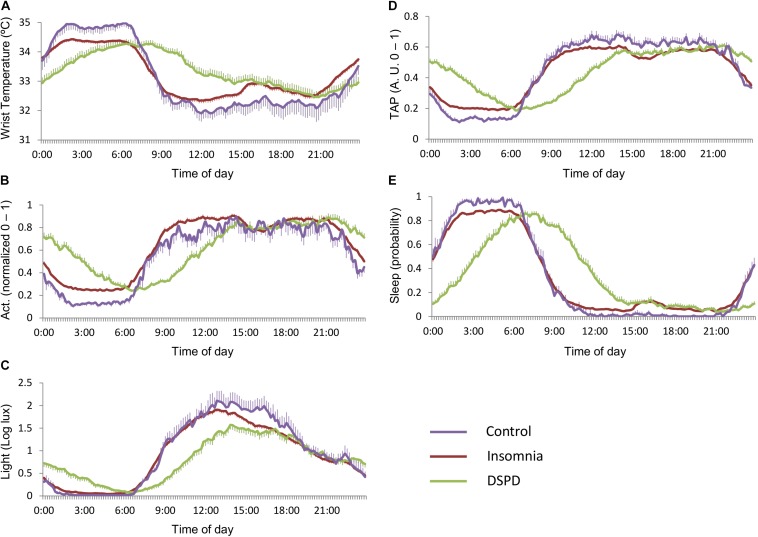
Mean circadian waveforms for **(A)** wrist temperature, **(B)** motor activity, **(C)** environmental light, **(D)** TAP, and **(E)** estimated sleep probability of the three main classes: healthy controls (purple), insomnia (red), and DSPD (green). Data are expressed as mean ± SEM.

Each class obtained by the decision tree was characterized by a specific circadian profile (Figure 3). Healthy controls presented: (a) deeper sleep: indicated by higher night wrist temperature and sleep probability, and lower motor activity and TAP levels during sleep, and (b) lower daytime temperature, pointing to higher activation levels than the remaining classes. DSPD was characterized by: (a) an evident delay of the nocturnal phase of all variables; (b) low activation during the morning (high temperature, low motor activity, and TAP); and (c) low levels of environmental light during the morning, with no differences with respect to the remaining classes from 17:00 to 23:00 h, and prolonged exposure to environmental light during the night (until 6:00 h, on average).

**FIGURE 4:**
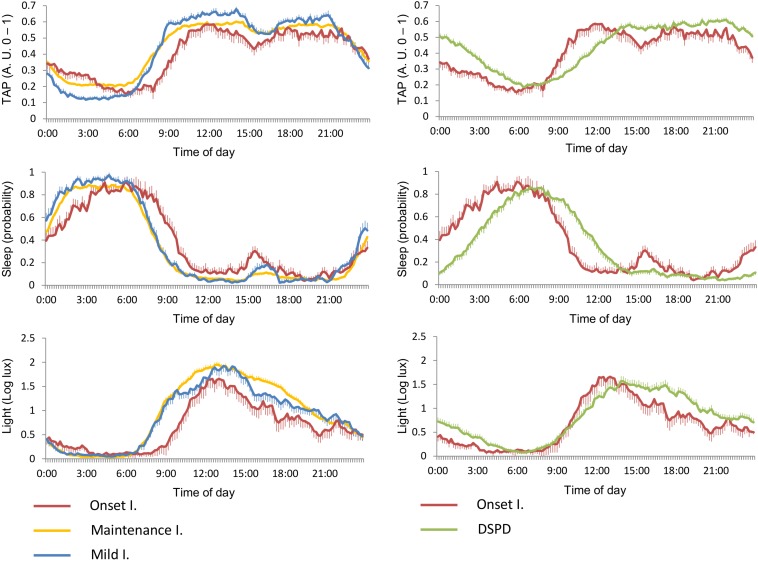
Mean circadian waveforms (from top to bottom) for TAP, estimated sleep, and exposure to environmental light rhythms, for insomnia subtypes **(left)** and onset insomnia vs. DSPD **(right)**. Data are expressed as mean ± SEM.

Figure 4, right panels, shows specific circadian profiles of the different subtypes of insomnia yielded by the model, labeled as onset, maintenance, or mild insomnia according to their characteristics. In consonance with the classification model, OI differed from the maintenance and mild insomnia by later sleep onset and wakeup times, while these two latter cases differed from each other in their TAP-RA. In the mild subtype, TAP showed lower night levels (lower general activation and, therefore, deeper sleep) and higher daytime levels (higher diurnal activation). Figure 4, left panels, shows specific circadian profiles of DSPD and OI. DSPD showed a sleep phase delay when compared to OI and, importantly, a different pattern of diurnal activation and exposure to light. In particular, OI exhibited higher general activation during the morning and more marked postprandial sleep propensity, while DSPD showed higher activation during the evening and early hours of the night. In addition, DSPD subjects were exposed to lower environmental light during the morning than the OI group, and higher levels after noon.

### Model Validation

**FIGURE 5:**
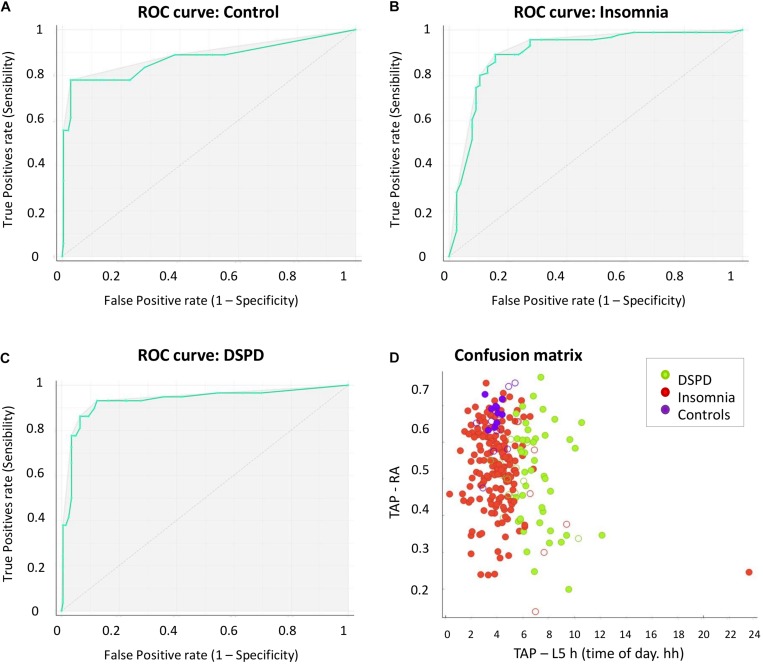
ROC curves for healthy controls **(A)**, insomnia **(B)**, and DSPD **(C)**. **(D)** The graphical representation of the confusion matrix data, as a function of the attributes TAP-RA (ranging from 0 to 1, *Y*-axis) and TAP-L5h (time of day, *X*-axis). Solid dots indicate true positives, and empty dots indicate false negatives. Cases classified as DSPD (green dots) were characterized by later mid-sleep time (TAP-L5h) than the other classes, while their TAP-RA scored within a wide range. Insomnia (red dots) exhibited a mid-sleep time between 3:00 and 5:00 h and, as in the case of DSPD, a wide range of TAP-RA scores. Healthy controls showed a mid-sleep time primarily centered around 4:00 h and predominantly high values of TAP-RA (0.65–0.7).

The model was evaluated through 10-fold cross-validation. Table 3 shows the accuracy, sensitivity, specificity, F1 index, and AUC of the model for each class, while false positive rates are displayed on the ROC curve, together with sensitivity (Figures 5A–C). This validation allowed for obtaining a confusion matrix, the data for which are shown in Figure 5D.

**TABLE 3:** Indexes for model validation.

**Class**	**Accuracy**	**Sensitivity**	**Specificity**	**F1**	**AUC**
Control	0.96	0.962	0.995	0.667	0.851
Insomnia	0.884	0.885	0.714	0.921	0.897
DSPD	0.922	0.923	0.964	0.811	0.934

## Discussion

The present study proposes a novel approach for clinically detecting sleep disorders using circadian markers. To this end, we evaluated the diagnostic potential of an ACM technique based on the simultaneous assessment of wrist temperature, motor activity, body position, and environmental light in a sample of patients suffering from either insomnia or DSPD, and a control group consisting of subjects without any circadian or sleep pathology. The potential of this technique for differential diagnostic was explored through machine learning analysis.

Machine learning methods are revolutionizing the field of clinical research as a support for differential diagnosis. They have recently been applied to widely different pathologies, such as diabetes (Dagliati et al., 2017), autism and attention-deficit hyperactivity disorder (ADHD) (Duda et al., 2017), glaucoma (Kim et al., 2017), Parkinson’s disease (Kubota et al., 2016), and pediatric inflammatory bowel disease (Mossotto et al., 2017).

To apply this technique in our study, we mainly based our analysis on the integrated variable TAP, obtained from wrist temperature, motor activity, and body position, since it showed higher accuracy for estimating sleep than any of the individual variables integrating it (Ortiz-Tudela et al., 2010, 2014). The circadian parameters of sleep estimated from TAP were also considered. A decision tree was built from just four of the indexes, calculated by non-parametric analyses (Refinetti et al., 2013). They included a sleep phase marker (TAP-L5h), a wakefulness phase marker (TAP-M10h), a global index of circadian rhythms robustness (sleep CFI), and TAP-RA. Besides quantifying the RA of the rhythms, which is a direct measure of rhythms quality, TAP-RA indirectly yields information on sleep depth and diurnal activation. Indeed, this index showed more discriminative power in our sample than the sleep depth estimators (TAP and motor activity L5v) themselves, probably because information on daytime is also considered.

Apart from discriminating among the three original categories, i.e., insomnia, DSPD, and healthy controls, the decision tree yielded three different groups of insomnia, which was likely facilitated by the large sample size and the consequent variability of the original insomnia group. One of them was characterized by a later mid-sleep time than the others, which pointed to sleep onset difficulties. Therefore, this subtype was labeled as OI. According to its late mid-sleep time, this insomnia subgroup emerged from the same branch as DSPD. The discrimination between these two pathologies thus relied on their central time of maximal activation, which was later in DSPD. This fits the expected circadian profile for DSPD, as it can be considered to be the clinical manifestation of extreme evening-types (Lack et al., 2009). In contrast, and according to our results, this would not be the case for OI, since it is not a circadian alteration. This finding is noteworthy, given the overlapping symptoms of these two pathologies (Gradisar and Crowley, 2013; Sivertsen et al., 2013; Richardson et al., 2015), especially when the diagnosis is based only on interviews. Our approach strongly supports the objective consideration of sleep–wake patterns and highlights the relevance of taking into account circadian rhythms when clinically evaluating sleep.

Interestingly, DSPD also differed from the other classes (including OI) in terms of its exposure rhythm to environmental light. In particular, it was characterized by null light exposure during the early hours of the morning, together with light exposure during a large part of the night. However, our model does not yield any information about causal relationships. Thus, we cannot infer whether their phase delay was partially a consequence or a cause of their light exposure pattern, since sleep–wake schedules modulate the light exposure schedules. In any case, it seems clear that there is a feedback between both factors and that this pattern of light exposure may contribute to maintaining the sleep and circadian alteration (Auger et al., 2011).

The subjects not showing delayed mid-sleep time were first divided by the RA of their rhythms, yielding another insomnia subgroup. This low contrast between sleep and wakefulness activation is congruent with fractioned sleep and daytime sleepiness. Therefore, this subgroup was considered to be compatible with MI. Among the remaining subjects, i.e., those with a high relative rhythm amplitude, another subgroup of insomnia emerged, which barely differed from healthy controls. The criterion used to differentiate between them was the robustness of the sleep rhythm, but their circadian profile in terms of the mean circadian waveforms was very similar. Thus, this subgroup of insomnia most likely consists of patients with low alteration, and it was therefore labeled as mild insomnia. It is important to note that our labeling of the insomnia subgroups was not meant as a diagnosis labeling, but as a characterization of the different groups obtained.

The most relevant limitation of our study was the large difference in the sample size of the initial categories and, specifically, the small sample size of healthy controls. This was due to the fact that participants were retrospectively selected from a clinical database, thus subjects suffering sleep and/or circadian disorders were more frequent than healthy controls. Such disparity of sample sizes may affect the quality of the model, as it would give more weight to the attributes that would better discriminate among the classes with larger sample size (in our case, insomnia), at the expense of the smaller classes, with the aim of maximizing the number of true positives. The most evident consequence of this limitation is the relatively high probability of misclassifying healthy participants as insomnia, in comparison with other groups. But this entails the risk of also increasing the rate of false positives in the largest group. In other words, the model would make decisions aimed at increasing sensitivity to insomnia, at the expense of specificity. Indeed, according to our results, the specificity for insomnia was the lowest, while it was very high in the other two categories (>95% in the case of DSPD and >99% in the case of the control group).

Despite this limitation, in general, our results showed high rates of sensitivity, accuracy, and specificity, thus confirming that our model was highly reliable for discriminating between the pathologies studied. However, future studies should address this limitation by applying this method to larger, and in particular, more balanced samples.

Another limitation would be that, since the present study follows a retrospective design, the device employed at the time of the data collection would result a little bit old fashioned nowadays. Nonetheless, it had previously shown to be highly useful and accurate for assessing circadian rhythms and sleep (Ortiz-Tudela et al., 2010, 2014). In fact, currently our Chronobiology Laboratory has implemented all temperature, motor activity, and environmental light sensors in a unique device, more modern and sophisticated that assesses simultaneously 15 different variables with a sampling rate of 10 Hz (Madrid-Navarro et al., 2019). It has been recently validated for Parkinson’s disease and allows also an accurate detection of the light type, intensity, and timing the subject is exposed under ambulatory conditions (Arguelles-Prieto et al., 2019).

So far, the results of the present study can be highly useful in clinical practice, since they permit a better characterization of insomnia and DSPD. Furthermore, our results inspire further research to address other classification models for various disorders, such as obstructive sleep apnea/hypopnea (OSAH) or advanced sleep phase disorder (ASPD). In addition, the validation of the ACM technique also encourages its application in research, as it would seem to be suitable for sample selection, evaluation, and even the monitoring of habits in order to offer recommendations (e.g., on light exposure and a sedentary lifestyle). The combination of wearable devices, which make it possible to record millions of data points per subject, and the development of methods based on massive data management and data mining (e.g., machine learning methods) facilitate “digital health,” providing new diagnostic tools and allowing personalized therapies in broad population samples and at an affordable cost.

## Data Availability Statement

The original datasets generated for this study are available on request to the corresponding author.

## Ethics Statement

The study was retrospective, included no intervention but the usual standard practice at the clinic. A personal dissociated database was used in order to comply with the Spanish Personal Data Protecting Law.

## Author Contributions

JM, MR, AC, and BR-M: study concept and design. EE, JA, and FS: acquisition of data. CE-D and BR-M: coordination and managing of database. MC, BR-M, and JM: analysis and interpretation of data. BR-M, JM, and MR: drafting of manuscript. BR-M, EE, CE-D, JA, FS, AC, JM, and MR: critical revision of the manuscript.

## Conflict of Interest

The authors declare that the research was conducted in the absence of any commercial or financial relationships that could be construed as a potential conflict of interest.

## Footnotes

1 https://kronowizard.um.es/kronowizard/inicio.seam

2 https://kronowizard.um.es/

## Abbreviations

ACM: ambulatory circadian monitoring
AUC: area under curve
CFI: circadian function index
DSPD: delayed sleep phase disorder
IS: interdaily stability
IV: intradaily variability
MI: maintenance insomnia
OI: onset insomnia
PI: primary insomnia
RA: relative amplitude.

## References

[B1] American Academy of Sleep Medicine (2005). *The International Classification of Sleep Disorders: Diagnostic & Coding Manual*, 2nd Edn, Westchester IL: American Academy of Sleep Medicine.

[B2] Ancoli-Israel S. Cole R. Alessi C. Chambers M. Moorcroft W. Pollak C. P. (2003). The role of actigraphy in the study of sleep and circadian rhythms. *Sleep* 26 342–392. 10.1093/sleep/26.3.342 12749557

[B3] Arguelles-Prieto R. Bonmati-Carrion M. A. Rol M. A. Madrid J. A. (2019). Determining light intensity, timing and type of visible and circadian light from an ambulatory circadian monitoring device. *Front. Physiol.* 10:822. 10.3389/fphys.2019.00822 31297069 PMC6607467

[B4] Auger R. R. Burgess H. J. Dierkhising R. A. Sharma R. G. Slocumb N. L. (2011). Light exposure among adolescents with delayed sleep phase disorder: a prospective cohort study. *Chronobiol. Int.* 28 911–920. 10.3109/07420528.2011.619906 22080736 PMC3405900

[B5] Bonmatí-Carrión M. Á Middleton B. Revell V. L. Skene D. J. Rol M. Á Madrid J. A. (2014). Circadian phase assessment by ambulatory monitoring in humans: correlation with dim light melatonin onset. *Chronobiol. Int.* 31 31–57. 10.3109/07420528.2013.820740 24164100

[B6] Borbély A. A. Daan S. Wirz-Justice A. Deboer T. (2016). The two-process model of sleep regulation: a reappraisal. *J. Sleep Res.* 25 131–143. 10.1111/jsr.12371 26762182

[B7] Dagliati A. Marini S. Sacchi L. Cogni G. Marsida T. Tibollo V. (2017). Machine learning methods to predict diabetes complications. *J. Diabetes Sci. Technol.* 12 295–302. 10.1177/1932296817706375 28494618 PMC5851210

[B8] Demšar J. Curk T. Erjavec A. Hočevar T. Milutinovič M. Možina M. (2013). Orange: data mining toolbox in python. *J. Mach. Learn. Res.* 14 2349–2353.

[B9] Duda M. Haber N. Daniels J. Wall D. P. (2017). Crowdsourced validation of a machine-learning classification system for autism and ADHD. *Transl. Psychiatr.* 7:e1133. 10.1038/tp.2017.86 28509905 PMC5534954

[B10] Fayyad U. M. Irani K. B. (1993). “Multi-interval discretization of continuous-valued attributes for classification learning,” in *Proceedings of the 13th International Joint Conference on Artificial Intelligence*, Chambery.

[B11] Goel N. Basner M. Rao H. Dinges D. F. (2014). Circadian rhythms, sleep deprivation, and human performance. *Prog. Mol. Biol. Transl. Sci.* 119 155–190. 10.1016/B978-0-12-396971-2.00007-5 23899598 PMC3963479

[B12] Gradisar M. Crowley S. J. (2013). Delayed sleep phase disorder in youth. *Curr. Opin. Psychiatr.* 26 580–585. 10.1097/YCO.0b013e328365a1d4 24060912 PMC4142652

[B13] Gradisar M. Gardner G. Dohnt H. (2011). Recent worldwide sleep patterns and problems during adolescence: a review and meta-analysis of age, region, and sleep. *Sleep Med.* 12 110–118. 10.1016/j.sleep.2010.11.008 21257344

[B14] Kim S. J. Cho K. J. Oh S. (2017). Development of machine learning models for diagnosis of glaucoma. *PLoS One* 12:e0177726. 10.5061/dryad.q6ft5 28542342 PMC5441603

[B15] Kubota K. J. Chen J. A. Little M. A. (2016). Machine learning for large-scale wearable sensor data in Parkinson’s disease: concepts, promises, pitfalls, and futures. *Mov. Disord.* 31 1314–1326. 10.1002/mds.26693 27501026

[B16] Lack L. Bailey M. Lovato N. Wright H. (2009). Chronotype differences in circadian rhythms of temperature, melatonin, and sleepiness as measured in a modified constant routine protocol. *Nat. Sci. Sleep* 1 1–8. 10.2147/NSS.S6234 23616692 PMC3630920

[B17] Liu H. Hussain F. (2002). Discretization: an enabling technique. *Data Min. Knowl. Disc.* 6 393–423.

[B18] Lovato N. Gradisar M. Short M. Dohnt H. Micic G. (2013). Delayed sleep phase disorder in an Australian school-based sample of adolescents. *J. Clin. Sleep Med.* 15 939–944. 10.5664/jcsm.2998 23997706 PMC3746721

[B19] Madrid-Navarro C. J. Javier F. Cuesta P. Escamilla-Sevilla F. Campos M. Abellán F. R. (2019). Validation of a device for the ambulatory monitoring of sleep patterns: a pilot study on Parkinson’s disease. *Front. Neurol.* 10:356. 10.3389/fneur.2019.00356 31031690 PMC6470193

[B20] Martinez-Nicolas A. Madrid J. A. García F. J. Campos M. Moreno-Casbas M. T. Almaida-Pagan P. F. (2018). Circadian monitoring as an aging predictor. *Sci. Rep.* 8:15027. 10.1038/s41598-018-33195-3 30301951 PMC6177481

[B21] Martinez-Nicolas A. Madrid J. A. Rol M. A. (2014). Day–night contrast as source of health for the human circadian system. *Chronobiol. Int.* 31 382–393. 10.3109/07420528.2013.861845 24304407

[B22] Martinez-Nicolas A. Martinez-Madrid M. J. Almaida-Pagan P. F. Bonmati-Carrion M. A. Madrid J. A. Rol M. A. (2019). Assessing chronotypes by ambulatory circadian monitoring. *Front. Physiol.* 10:1396 10.3389/fphys.2019.01396 PMC6879660 31824327

[B23] Martinez-Nicolas A. Ortiz-Tudela E. Madrid J. A. Rol M. A. (2011). Crosstalk between environmental light and internal time in humans. *Chronobiol. Int.* 28 617–629. 10.3109/07420528.2011.593278 21793693

[B24] Martinez-Nicolas A. Ortiz-Tudela E. Rol M. Á Madrid J. A. (2013). Uncovering different masking factors on wrist skin temperature rhythm in free-living subjects. *PLoS One* 8:e61142. 10.1371/journal.pone.0061142 23577201 PMC3618177

[B25] Maslove D. M. Podchiyska T. Lowe H. J. (2013). Discretization of continuous features in clinical datasets. *J. Am. Med. Inform. Assoc.* 20 544–553. 10.1136/amiajnl-2012-000929 23059731 PMC3628044

[B26] Mossotto E. Ashton J. J. Coelho T. Beattie R. M. MacArthur B. D. Ennis S. (2017). Classification of paediatric inflammatory bowel disease using machine learning. *Sci. Rep.* 7:2427. 10.1038/s41598-017-02606-2 28546534 PMC5445076

[B27] Mullington J. M. Abbott S. M. Carroll J. E. Davis C. J. Dijk D.-J. Dinges D. F. (2016). Developing biomarker arrays predicting sleep and circadian-coupled risks to health. *Sleep* 39 727–736. 10.5665/sleep.5616 26951388 PMC4791606

[B28] Ortiz-Tudela E. Martinez-Nicolas A. Albares J. Segarra F. Campos M. Estivill E. (2014). Ambulatory circadian monitoring (ACM) based on thermometry, motor activity and body position (TAP): a comparison with polysomnography. *Physiol. Behav.* 126 30–38. 10.1016/j.physbeh.2013.12.009 24398067

[B29] Ortiz-Tudela E. Martinez-Nicolas A. Campos M. Rol M. Á Madrid J. A. (2010). A new integrated variable based on thermometry, actimetry and body position (TAP) to evaluate circadian system status in humans. *PLoS Comput. Biol.* 6:e1000996. 10.1371/journal.pcbi.1000996 21085644 PMC2978699

[B30] Owens J. Maxim R. McGuinn M. Nobile C. Msall M. Alario A. (1999). Television-viewing habits and sleep disturbance in school children. *Pediatrics* 104:e27. 10.1542/peds.104.3.e27 10469810

[B31] Refinetti R. Lissen G. C. Halberg F. (2013). Procedures for numerical analysis of circadian rhythms. *Biol. Rhythm Res.* 38 275–325. 10.1080/09291010600903692 23710111 PMC3663600

[B32] Richardson C. E. Gradisar M. Barbero S. C. (2015). Are cognitive “insomnia” processes involved in the development and maintenance of delayed sleep wake phase disorder? *Sleep Med. Rev.* 26 1–8. 10.1016/j.smrv.2015.05.001 26140864

[B33] Roenneberg T. Wirz-Justice A. Merrow M. (2003). Life between clocks: daily temporal patterns of human chronotypes. *J. Biol. Rhythm.* 18 80–90. 10.1177/0748730402239679 12568247

[B34] Rokach L. Maimon O. (2005). Top-down induction of decision trees classifiers—a survey. *IEEE Trans. Syst. Man Cyber.* 35 476–487. 10.1109/TSMCC.2004.84324731

[B35] Roth T. (2007). Insomnia: definition, prevalence, etiology, and consequences. *J. Clin. Sleep Med.* 3(5 Suppl.), 3–6. 10.1378/chest.14-0970 17824495 PMC1978319

[B36] Sadeh A. Acebo C. (2002). The role of actigraphy in sleep medicine. *Sleep Med. Rev.* 6 113–124. 10.1053/smrv.2001.0182 12531147

[B37] Sarabia J. A. Rol M. Á Mendiola P. Madrid J. A. (2008). Circadian rhythm of wrist temperature in normal-living subjects. A candidate of new index of the circadian system. *Physiol. Behav.* 95 570–580. 10.1016/j.physbeh.2008.08.005 18761026

[B38] Schrader H. Bovim G. Sand T. (1993). The prevalence of advanced and delayed sleep phase syndromes. *J. Sleep Res.* 2 51–55. 10.1111/j.1365-2869.1993.tb00061.x 10607071

[B39] Serrano J. I. Romero J. P. Castillo M. D. del Rocon E. Louis E. D. Benito-León J. (2017). A data mining approach using cortical thickness for diagnosis and characterization of essential tremor. *Sci. Rep.* 7:2190. 10.1038/s41598-017-02122-3 28526878 PMC5438396

[B40] Shochat T. (2012). Impact of lifestyle and technology developments on sleep. *Nat. Sci. Sleep* 4 19–31. 10.2147/NSS.S18891 23616726 PMC3630968

[B41] Shochat T. Flint-Bretler O. Tzischinsky O. (2010). Sleep patterns, electronic media exposure and daytime sleep-related behaviours among Israeli adolescents. *Acta Paediatr. Int. J. Paediatr.* 99 1396–1400. 10.1111/j.1651-2227.2010.01821.x 20377536

[B42] Sivertsen B. Pallesen S. Stormark K. M. Bøe T. Lundervold A. J. Hysing M. (2013). Delayed sleep phase syndrome in adolescents: prevalence and correlates in a large population based study. *BMC Public Health* 13:1163. 10.1186/1471-2458-13-1163 24330358 PMC3878844

[B43] Van Den Bulck J. (2004). Television viewing, computer game playing, and internet use and self-reported time to bed and time out of bed in secondary-school children. *Sleep* 27 101–104. 10.1093/sleep/27.1.101 14998244

[B44] Weitzman E. Czeisler C. Coleman R. Spielman A. Zimmerman J. Dement W. (1981). Delayed sleep phase syndrome: a chronobiological disorder with sleep-onset insomnia. *Arch. Gen. Psychiatry* 38 737–746. 7247637 10.1001/archpsyc.1981.01780320017001

[B45] World Health Organization [WHO] (2008). *International Statistical Classification of Diseases and Related Health Problems.* Geneva: World Health Organization.

[B46] Yazaki M. Shirakawa S. Okawa M. Takahashi K. (1999). Demography of sleep disturbances associated with circadian rhythm disorders in Japan. *Psychiatr. Clin. Neurosci.* 53 267–268. 10.1046/j.1440-1819.1999.00533.x 10459707

[B47] Zornoza-Moreno M. Fuentes-Hernández S. Prieto-Sánchez M. T. Blanco J. E. Pagán A. Rol M. -Á, et al. (2013). Influence of gestational diabetes on circadian rhythms of children and their association with fetal adiposity. *Diabetes Metab. Res. Rev.* 29 483–491. 10.1002/dmrr.2417 23568539

